# Emergence of CD134 cysteine-rich domain 2 (CRD2)-independent strains of feline immunodeficiency virus (FIV) is associated with disease progression in naturally infected cats

**DOI:** 10.1186/s12977-014-0095-7

**Published:** 2014-11-28

**Authors:** Paweł M Bęczkowski, Navapon Techakriengkrai, Nicola Logan, Elizabeth McMonagle, Annette Litster, Brian J Willett, Margaret J Hosie

**Affiliations:** MRC-University of Glasgow Centre for Virus Research, Glasgow, UK; Small Animal Hospital, University of Glasgow, Glasgow, UK; Department of Veterinary Clinical Sciences, Purdue University, West Lafayette, IN 47907 USA

**Keywords:** FIV, CD134, OX40, CRD2, Receptor, Evolution, Natural infection, Disease progression

## Abstract

**Background:**

Feline immunodeficiency virus (FIV) infection is mediated by sequential interactions with CD134 and CXCR4. Field strains of virus vary in their dependence on cysteine-rich domain 2 (CRD2) of CD134 for infection.

**Findings:**

Here, we analyse the receptor usage of viral variants in the blood of 39 naturally infected cats, revealing that CRD2-dependent viral variants dominate in early infection, evolving towards CRD2-independence with disease progression.

**Conclusions:**

These findings are consistent with a shift in CRD2 of CD134 usage with disease progression.

**Electronic supplementary material:**

The online version of this article (doi:10.1186/s12977-014-0095-7) contains supplementary material, which is available to authorized users.

## Findings

The interaction between a virus and its cellular receptor is a primary event in viral replication and the specificity of the virus-receptor interaction influences both viral cell tropism and pathogenicity. While the primate lentiviruses target helper T cells by binding to CD4 [1-3], the feline immunodeficiency virus (FIV) attaches to helper T cells through an interaction with CD134 (OX40) [4,5]. Infection with either the primate or the feline lentiviruses requires a subsequent interaction with a member of the seven transmembrane domain (7TM) superfamily molecules; for the human immunodeficiency virus (HIV) the major co-receptors are CXCR4 and CCR5 [6-10], while FIV uses CXCR4 as a sole co-receptor [11,12].

Assessment of HIV-1 cell tropism may be used to monitor disease progression [13,14] because progression is often associated with the emergence of either X4 viruses or dual-tropic viruses capable of using both CXCR4 and CCR5, (“X4R5” viruses) [15]. The emergence of X4 variants is usually accompanied by a rapid decline in the number of CD4^+^ T cells and the onset of immunodeficiency [16] and X4 or X4R5 viruses are present in 40-50% of HIV-1-infected individuals who progress to AIDS [17-20]. Like HIV, the cell tropism of FIV alters with disease progression; while CD4^+^ T cells are the predominant target in the acute (early) phase of infection, in the chronic (late) phase of infection, FIV also targets CD8^+^ T cells and B cells [21,22]. This shift in cell tropism between early and late viruses would seem counter-intuitive given that FIV infection is entirely dependent on sequential interactions between Env and CD134 [4,5,23] and CXCR4 [11,12,23-26]. Resolution of this enigma is offered by the observation that distinct strains of FIV differ in the nature of their interactions with CD134 [27-30]. It has been proposed that viruses requiring only CRD1 of CD134 for attachment (CRD2-independent strains) emerge during the later stages of disease [30,31]. In contrast, viruses that dominate in early infection require both CRD1 and CRD2 of CD134 for attachment (CRD2-dependent) [30,31]. The selective pressure for the emergence of CRD2-independent viruses may be escape from humoral or cellular immunity, with the accrued mutations in Env impacting on the virus-receptor interaction [30,32]. Consistent with this hypothesis, when cats were challenged with a reconstituted viral quasispecies containing equal titres of clonal variants with either CRD2-dependent or CRD2-independent Envs (early and late phenotypes), the CRD2-dependent viruses expanded preferentially *in vivo* while the CRD2-independent viruses failed to thrive [33].

## Methods

In this study, we examined the phenotype of viral variants isolated from outbred, privately owned US cats with naturally acquired FIV infection [34]. Forty-four cats were enrolled in the study on the basis of a history of FIV infection (confirmed by virus isolation), regardless of breed, sex, age and health status. Four blood samples (denoted A, B, C and D) were collected from each cat at 6-monthly intervals over an 18-month period, unless cats died during the interim period. The data recorded every 6 months included signalment, clinical history, physical examination data and body weight, as well as flow cytometric analyses of CD4^+^ and CD8^+^ T cells [34].

Full-length FIV *env* genes were amplified from whole blood using a two-step nested PCR protocol followed by direct nucleic acid sequence determination [34]. The nucleic acid sequence of the first-round PCR product informed primer design for the second round PCR; strain-specific primers [see Additional file 1: Table S1] for second round PCR incorporated restriction sites to facilitate sub-cloning into the expression vector for pseudotyping onto HIV. HIV(FIV)*luc* pseudotypes were prepared as described previously [4] by co-transfection of each Env construct with pNL4-3-Luc-E^−^R^−^luc into HEK-293 T cells. Pseudotype-containing culture fluids were harvested 72 hours post-transfection and stored frozen at −80°C until required.

The CRD2-dependence of each pseudotype was determined using assays for receptor usage of FIV Envs using MCC cells [35] expressing human (HHH), feline (FFF), and a feline x human chimaeric CD134 molecule containing human CRD2 (FFHH), as described previously [27,29].

Sequential plasma samples from infected cats were diluted ten-fold from 1 in 10 to 1 in 10,000 in complete RPMI 1640 medium (Life Technologies), before testing for the presence of homologous and heterologous neutralising antibodies (NAb) using HIV(FIV)luc pseudotypes as described previously [36].

## Results

### FIV Envs displayed a broad spectrum of cell tropism in naturally infected cats

**Figure 1:**
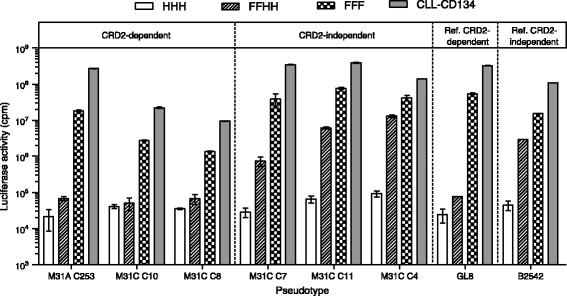
**Representative example of results obtained from receptor utilisation assay testing six autologous Env variants isolated from cat M31 and two reference Envs, GL8 (CRD2-dependent) and B2542 (CRD2-independent).** The mean luciferase counts per minute (cpm) for each cell line with standard errors (n = 3) are presented for MCC cells [35] expressing the entire human CD134 molecule (HHH, open bars), cells expressing the human x feline CD134 containing the human CRD2 domain (FFHH, hatched bars) and cells expressing the entire feline CD134 molecule (FFF and CLL-CD134 cells [29], shown in chequered and grey bars respectively). Note the differences in titres on cells expressing the human x feline CD134 chimaera containing the human CRD2 domain (FFHH cells, hatched bars) and subsequent pseudotype classification as CRD2-dependent or –independent.

While we cannot exclude the possibility that other variants existed in the cats, the methodology used provided a snapshot of the spectrum of Env variants present at each time point. The HIV(FIV) pseudotypes bearing FIV Envs derived from 39 (39/44, 88.6%) of the infected cats displayed a broad spectrum of cell tropisms (viable pseudotypes bearing Envs from 5 cats were not produced, predominantly because premature stop codons occurred in the *env* sequences obtained from these cats). Of the 287 Envs tested, 222 (77.4%) were CRD2-dependent, utilising CD134 in the same manner as the prototypic “early” isolate, GL-8 [Figure 1 and Table 1]. In contrast, 49 (17.0%) Envs were CRD2-independent, similar to the representative “late” isolate, B2542. The remaining 16 Envs (5.6%) displayed an intermediate pattern of receptor usage. Some cats (for example P4, M5 and M31) harboured a mixture of CRD2-dependent and -independent Env variants.

**Table 1:** **Mode of CD134 utilisation displayed by 287 Envs from 39 cats**

**Cat**	**Alive/Dead**	**Health status**	**CD4 count**	**Mode of CD134 interaction**			**No. of Envs tested**	**Homologous neutralisation**
				**CRD2-dependent**	**CRD2-independent**	**Intermediate**		
M28	A	H	> 350	11 (100%)	0	0	11	-
M47	A	H	< 350	8 (100%)	0	0	8	+
M8	A	S	< 350	2 (100%)	0	0	2	-
P9	A	S	< 350	6 (100%)	0	0	6	+
M1	A	H	< 350	4 (100%)	0	0	4	-
M2	A	H	> 350	2 (100%)	0	0	2	+
M29	A	H	> 350	3 (100%)	0	0	3	-
P1	A	H	> 350	3 (100%)	0	0	3	-
P11	A	H	> 350	7 (100%)	0	0	7	+
P14	A	H	> 350	9 (100%)	0	0	9	+
P18	A	H	> 350	1 (100%)	0	0	1	+
P21	A	H	> 350	6 (100%)	0	0	6	-
P22	A	H	> 350	2 (100%)	0	0	2	+
P4	A	H	< 350	4 (44.4%)	2 (22.2%)	3 (33.3%)	9	+
P5	A	H	< 350	7 (87.5%)	1 (12.5%)	0	8	+
P6	A	H	> 350	8 (100%)	0	0	8	-
M15	A	S	> 350	6 (66.6%)	3 (33.3%)	0	9	+
M20	A	S	> 350	1 (100%)	0	0	1	-
M32	A	S	< 350	3 (75%)	1 (25%)	0	4	-
P13	A	S	< 350	8 (88.9%)	1 (11.1%)	0	9	+
P17	A	S	< 350	9 (100%)	0	0	9	-
P7	A	S	> 350	1 (50%)	0	1 (50%)	2	+
P8	A	S	> 350	5 (100%)	0	0	5	-
M25	D	H	< 350	3 (50%)	2 (33.3%)	1 (16.7%)	6	+
M46	D	H	< 350	7 (87.5%)	1 (12.5%)	0	8	+
M49	D	H	< 350	6 (100%)	0	0	6	+
M14	D	S	< 350	9 (90%)	1 (10%)	0	10	+
M26	D	S	< 350	4 (66.7%)	2 (33.3%)	0	6	+
M30	D	S	< 350	15 (94%)	1 (6%)	0	16	+
M33	D	S	< 350	2 (11%)	17 (89%)	0	19	-
M41	D	S	< 350	9 (75%)	2 (17%)	1 (8%)	12	-
M5	D	S	< 350	5 (55.6%)	3 (33.3%)	1 (11.1%)	9	-
M11	D	H	> 350	17 (81%)	3 (14%)	1 (5%)	21	-
M50	D	H	> 350	2 (100%)	0	0	2	-
P2	D	H	> 350	16 (100%)	0	0	16	+
M16	D	S	> 350	0	1 (14.3%)	6 (85.7%)	7	-
M3	D	S	< 350	0	1 (50%)	1 (50%)	2	-
M31	D	S	< 350	9 (53%)	7 (41%)	1 (6%)	17	+
M44	D	S	> 350	2 (100%)	0	0	2	+

Cats were classified according to their clinical status at the time of enrolment (H = clinically healthy, S = sick), CD4^+^ cell counts (< or >350 cells/μl) and survival during the study period (A = alive, D = deceased). For absolute CD4^+^ T cell counts see Additional file 2 [Table S2]. Mode of CD134 interaction: numbers (and percentages) of Envs classified as: 1) CRD2-dependent; 2) CRD2-independent; 3) intermediate requirement for determinants within CRD2. For homologous neutralisation, “+” and “-” indicate ≥ or <2.5 fold neutralisation, respectively. For complete neutralisation data see Additional file 3 [Table S3].

### CRD2-independence segregates with clinical status

**Figure 2:**
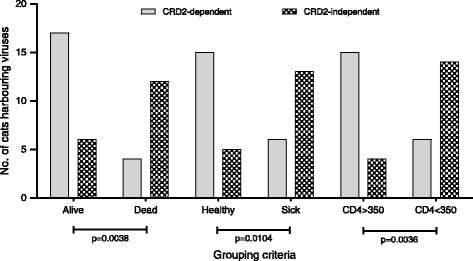
**CRD2-independent Env variants were isolated more frequently from cats displaying signs of immunodeficiency and disease progression.** Cats were classified into 3 groups: 1) alive/deceased, 2) clinically healthy/sick, 3) CD4^+^ T cell counts > or <350 cells/μl. Cats which died during the study, displayed clinical signs (sick) or had CD4^+^ T cell counts <350 cells/μl were more likely to harbour at least one CRD2-independent variant (chequered bars). Conversely, CRD2-dependent variants were associated with the alive, clinically healthy cats with CD4^+^ T cell counts >350 cells/μl (grey bars). P-values (Fisher’s exact test) were shown below the x-axis.

Since CRD2-independent variants of FIV were present in only a subset of the cats studied, we examined the data for evidence of an association between clinical status and the presence of CRD2-independent variants. Since the duration of infection was unknown, cats were grouped retrospectively according to their status at time of last sampling: Alive or Dead; Clinically Healthy (no clinical abnormalities detected on physical examination) or Sick (at least one clinical abnormality detected); a CD4 count >350 cells/μl or <350 cells/μl. Statistical analysis of viral load data revealed no significant differences between cats that were classified as clinically healthy or sick at the time of enrolment (data not shown). When the number of cats giving rise to CRD2-independent Env variants was compared between groups, a strong association was noted between the presence of CRD2-independent variants and a decline in clinical status [Figure 2]. Samples collected from cats that died during the study had a significantly higher proportion of CRD2-independent variants than samples from cats that were alive at the time of last sampling (p = 0.0038, Fisher’s exact test). Similarly, sick cats (displaying clinical signs) harboured more CRD2-independent viruses than healthy cats (p = 0.0104, Fisher’s exact test).

Given that a clinical assessment of health status may be subjective, we next used a CD4 count <350 cells/μl as indicative of CD4^+^ T cell depletion and progression to immunodeficiency (by analogy to WHO recommendations for the initiation of antiretroviral therapy for HIV infection [http://www.who.int/hiv/pub/guidelines/arv2013/en/]), for data see Additional file 2: Table S2. We observed that immunodeficient cats were more likely to harbour CRD2-independent Envs than cats with CD4 counts >350 cells/μl (p = 0.0036, Fisher’s exact test). Thus the presence of CRD2-independent viruses was associated with declining clinical status.

### Absence of correlation between CRD2-usage and neutralising antibody response

When neutralisation profiles [see Additional file 3: Table S3] were compared [Table 1], plasma from 10/17 cats harbouring at least one CRD2-independent Env variant neutralised pseudotypes bearing homologous Envs, while the remaining 7 cats showed no evidence of homologous neutralisation (p = 0.75, Fisher’s exact test). Thus, no correlation was observed between the presence of neutralising antibodies and CRD2-independence, indicating that key factors driving Env evolution remain to be discovered.

### Loss of predicted N-linked glycosylation site was associated with decrease in CRD2 dependence

A complete spectrum of receptor utilisation was observed by pseudotypes bearing 21 Env variants isolated from three time points from cat M11 (until its death); 17/21 were CRD2-dependent, 3/21 were CRD2-independent, and one displayed an intermediate phenotype [Table 1]. Strikingly, the remaining pseudotype (M11A C242) infected cells expressing the CD134 chimaera containing the human CRD2 domain as efficiently as cells expressing the entire feline CD134 molecule [see Additional file 4: Figure S1]. Analysis of amino acid sequence alignments identified two unique mutations: 1) T520A and 2) E838K which were present in M11A C242 Env but not any other Env variants from this cat. The T520A substitution led to the loss of a predicted site for N-linked glycosylation (PNGS), consistent with a role for the T520A mutation in the observed decrease in CRD2 dependence. To confirm this observation, PCR-based site directed mutagenesis was employed to exchange 1) alanine at residue 520 to threonine (A520T) and 2) lysine at residue 838 to glutamate (K838E), creating two mutants, namely M11A C242 T520 and M11A C242 E838 respectively. By comparing the receptor utilisation of these mutant Envs with the wild type M11A C242 Env, we observed that the M11A C242 T520 mutant achieved a 10 fold lower titre on cells expressing the human x feline CD134 chimaera containing the human CRD2 domain compared to either the parental wild type M11A C242 or the M11A C242 E838 mutant Env, consistent with residue A520 in the wild type Env (ablating the PNGS) having an important role in receptor utilisation [see Additional file 4: Figure S1].

To confirm the role of A520 and the subsequent loss of a PNGS in determining receptor utilisation, mutants of the Env clone M11C C164, (CRD2-dependent phenotype) were constructed. Substitution of T520 with A520 resulted in the generation of mutant M11C C164 A520 that productively infected cells expressing the human x feline CD134 chimaera containing the human CRD2 domain [see Additional file 4: Figure S1], consistent with a role for the PNGS at residue 520 in modulating CRD2-dependence and altering the virus-receptor interaction.

The envelope glycoprotein adopts a trimeric structure upon the virion and it is likely that multiple determinants within Env may affect the tertiary or quaternary structure of the trimer, resulting in altered CD134 utilisation. The data presented here are consistent with previous studies [29,32] indicating that single mutations within the V1/V2 or V5 regions of Env alone, likely as a result of pressure from the host immune response, may alter the structure of Env sufficiently to drive the emergence of CD134 CRD2-independent variants of FIV. Whether the emergence of such variants is a cause or consequence of disease progression remains to be established.

## Conclusion

The studies described herein are consistent with the dominance of CRD2-dependent viruses in early infection and indicate that the emergence of CRD2-independent viruses coincides with clinical decline towards immunodeficiency. Accordingly, viral phenotyping using HIV(FIV) pseudotypes may assist with the clinical staging of FIV infection. Moreover, if CRD2-dependent viruses dominate in early infection, testing prototypic FIV vaccines against challenge with CRD2-dependent strains of virus will likely predict vaccine efficacy in the field.

## Additional files

Additional file 1: Table S1. Strain specific primer sequences. Additional file 2: Table S2. CD4^+^ T cell counts (cells/μl) for each time point. Additional file 3: Table S3. Mode of CD134 utilisation for each Env pseudotype. Additional file 4: Figure S1. PNGS at the stem of V5 region of Env is associated with distinct mode of receptor utilisation.
