# Supplementary material for: Emergence of CD134 cysteine-rich domain 2 (CRD2)-independent strains of feline immunodeficiency virus (FIV) is associated with disease progression in naturally infected cats
Source: Retrovirology. 2014 Nov 28;11:95. doi: 10.1186/s12977-014-0095-7 (PMC4275942; doi:10.1186/s12977-014-0095-7)
Supplement: Additional file 1: Table S1. — Strain specific primer sequences. [file 12977_2014_95_MOESM1_ESM.docx]

**Additional file 1: Table S1** Strain specific primer sequences

| **Primer** | **Sequence (5'-3')** | **Purpose** |
| --- | --- | --- |
| 2F2 | TATTATTGGCARTTGCAATCTACMTTATC | Blood direct PCR forward primer |
| 1R4 | CCAATAMTCWTCCCAGTCCACCCTT | Blood direct PCR reverse primer |
| G8_F | GGGTCGACACCATGGCAGAAGGGTTTGCAGCA | Second round forward primer |
| M9M12_F | ACTGGTCGACACCATGGCAGCAGGGAGGATTTACTCAA | Second round forward primer |
| M14M25_F | ACTGGTCGACACCATGGCAGAGGGAGGATTTACTCAA | Second round forward primer |
| M8M33M43_F | ACTGGTCGACACCATGGCAGAAGGATTTGCAGCC | Second round forward primer |
| M30M31M49_F | ACTGGTCGACACCATGGCAGAGGGAGGATTTACTCAA | Second round forward primer |
| M47M50_F | ACTGGTCGACACCATGGCAGAAGGATTTGCAGTC | Second round forward primer |
| M6_F | ACTGGTCGACACCATGGCAGAGGGAGGATTTGCTCAA | Second round forward primer |
| M2_F | ACTGGTCGACACCATGGCAGAAAGATTTGCAGCC | Second round forward primer |
| M11_F | ACTGGTCGACACCATGGCAGAGGGAGGATTTAATCAA | Second round forward primer |
| M46_F | ACTGGTCGACACCATGGCAGAGGGAGGATTTACTCAG | Second round forward primer |
| M48_F | ACTGGTCGACACCATGGCAGAAGGATTCACTCAA | Second round forward primer |
| P4P5P13P15_F | ACTGGTCGACACCATGGCAGAAGGATTTGCAGCC | Second round forward primer |
| P2P6P11P14P17_F | ACTGGTCGACACCATGGCAGAGGGAGGATTTACTCAA | Second round forward primer |
| P10_F | ACTGGTCGACACCATGGCAGAGGGAAGGTTTACTCAA | Second round forward primer |
| M6_R | ACAGCGGCCGCCATCATTTCTCCTCTTTTTCAGAC | Second round reverse primer |
| M31_R | ACAGCGGCCGCGATCATTCCTCCTCTTTTTCAGAC | Second round reverse primer |
| M9M25M33M43M46_R | ACAGCGGCCGCCATCATTCCTCCTCTTTTTCAGAC | Second round reverse primer |
| M14M30M48_R | ACAGCGGCCGCCATCATTCCTCCTCTTTTTCAGAT | Second round reverse primer |
| M47_R | ACAGCGGCCGCCATCATTCCTCCTCTTTTTCAGGT | Second round reverse primer |
| P2P5P13_R | ACAGCGGCCGCCATCATTCCTCCTCTTTTTCAGAT | Second round reverse primer |
| P14_R | ACAGCGGCCGCCATCATTCCTCCTCTTTTTCAGAC | Second round reverse primer |
| P6P7_R | GGGCGGCCGCCATCGCCTCCTCTTTTTCAGA | Second round reverse primer |
| G8_R | GGGCGGCCGCCATCATTCCTCCTCTTTTTCAGAC | Second round reverse primer |
| VR1012_F | CTTTTCTGCAGTCACCGTCG | Sequencing forward primer |
| VR1012_R | CTGGATCCAGGCGCCTGGTCTA | Sequencing reverse primer |
| Memphis780_F | TGGAATGAGACTATAACAGG | Sequencing forward primer |
| Memphis2090_R | GATTACATCCTAATTCTTGCATAG | Sequencing reverse primer |
