# Supplementary material for: Emergence of CD134 cysteine-rich domain 2 (CRD2)-independent strains of feline immunodeficiency virus (FIV) is associated with disease progression in naturally infected cats
Source: Retrovirology. 2014 Nov 28;11:95. doi: 10.1186/s12977-014-0095-7 (PMC4275942; doi:10.1186/s12977-014-0095-7)
Supplement: Additional file 2: Table S2. — CD4+ T cell counts (cells/μl) for each time point. [file 12977_2014_95_MOESM2_ESM.docx]

**Additional file 2: Table S2** CD4^+^ T cell counts (cells/µl) for each time point (A, B, C and D) unless cat was deceased (+) or sample was not available (NA). ΔCD4 in the final column represents the difference between the first (A) and the last available sampling.

| Cat | CD4 count (cells/µl) | | | | ΔCD4 (cells/µl) | Cat | CD4 count (cells/µl) | | | | ΔCD4 (cells/µl) |
| --- | --- | --- | --- | --- | --- | --- | --- | --- | --- | --- | --- |
|  | Time point | | | |  |  | Time point | | | |  |
|  | A | B | C | D |  |  | A | B | C | D |  |
| M2 | 1740 | 360 | 760 | 620 | -1120 | M5 | 560 | 150 | 160 | + | -400 |
| M29 | 1480 | 620 | 930 | 620 | -860 | M50 | 1380 | 1320 | + | + | -70 |
| M1 | 870 | 360 | 340 | 260 | -610 | M33 | 200 | N/A | + | + | N/A |
| M15 | 870 | 1030 | 1090 | 470 | -400 | M3 | 330 | + | + | + | N/A |
| M8 | 550 | 210 | 200 | 150 | -400 | M44 | 1240 | + | + | + | N/A |
| M49 | 410 | 310 | 270 | 40 | -370 | P4 | 90 | 340 | 330 | NA | 240 |
| M28 | 1230 | 890 | 1770 | 900 | -330 | P14 | 970 | 1160 | 1090 | NA | 120 |
| M14 | 450 | 290 | 150 | 140 | -310 | P8 | 500 | 860 | 600 | NA | 100 |
| M25 | 360 | 390 | 680 | 90 | -270 | P6 | 480 | 350 | 570 | NA | 90 |
| M20 | 1500 | 810 | NA | 1250 | -250 | P7 | 400 | 260 | 400 | NA | 0 |
| M47 | 290 | 140 | 140 | 100 | -190 | P11 | 450 | 350 | 450 | NA | 0 |
| M32 | 380 | 320 | 350 | 210 | -170 | P13 | 460 | 280 | 270 | NA | -190 |
| M30 | 130 | 150 | 260 | 100 | -30 | P17 | 490 | 550 | 280 | NA | -210 |
| M46 | 150 | 190 | 180 | 180 | 30 | P9 | 630 | 350 | 180 | NA | -450 |
| M11 | 980 | 140 | 480 | + | -500 | P5 | 790 | 720 | 300 | NA | -490 |
| M16 | 350 | 370 | 360 | + | 10 | P2 | 400 | 360 | + | + | -40 |
| M26 | 540 | 140 | 130 | + | -410 | P21 | NA | 930 | 570 | NA | -360 |
| M31 | 800 | 880 | 260 | + | -540 | P22 | NA | 1550 | 790 | NA | -760 |
| M41 | 340 | 350 | 120 | + | -220 | P18 | NA | 730 | 750 | NA | 20 |
