# Supplementary material for: Emergence of CD134 cysteine-rich domain 2 (CRD2)-independent strains of feline immunodeficiency virus (FIV) is associated with disease progression in naturally infected cats
Source: Retrovirology. 2014 Nov 28;11:95. doi: 10.1186/s12977-014-0095-7 (PMC4275942; doi:10.1186/s12977-014-0095-7)
Supplement: Additional file 3: Table S3. — Mode of CD134 utilisation for each Env pseudotype. [file 12977_2014_95_MOESM3_ESM.docx]

**Additional file 3: Table S3** Mode of CD134 utilisation for each Env pseudotype (CRD2-independence denoted as “+”, CRD2-dependence as “-”, and intermediate mode of interaction with CRD2 as “+/-”). Receptor utilisation data are shown in relation to autologous and heterologous neutralising antibody (NAb) responses of plasma samples expressed as fold neutralisation (fold neutralisation was calculated by dividing the mean luciferase counts of control wells containing no plasma with the mean luciferase counts for wells containing 1 in 10 plasma dilutions). Sequential plasma samples from 38 cats (plasma sample from one cat (1/39) was exhausted and not available for analysis) were assessed against pseudotypes bearing autologous Envs and two reference pseudotypes, bearing Envs of GL-8 and B2542 (shown below each set of autologous pseudotypes for each respective cat). Plasma samples collected at 6 monthly intervals (A, B, C and D) expressing weak, moderate and strong neutralisation are highlighted in yellow, orange and red respectively. Plasma samples that failed to neutralise are shown in background colour. The number of PNGS is shown next to the specific FIV Env variant assessed for its susceptibility to neutralisation. (NA-not available, PNGS-potential N-linked glycosylation sites).

| **FIV Env** | **PNGS** | **Plasma collected at sequential time points** | | | | **CRD2 independence** |  | **FIV Env** | **PNGS** | **Plasma collected at sequential time points** | | | | **CRD2 independence** |
| --- | --- | --- | --- | --- | --- | --- | --- | --- | --- | --- | --- | --- | --- | --- |
|  |  | A | B | C | D |  |  |  |  | A | B | C | D |  |
| M15A C13 | 25 | 1 | 1 | 1 | 1 | - |  | P14A C25 | 24 | 303 | 381 | 357 | 356 | - |
| M15A C42 | 25 | 2483 | 2594 | 1744 | 2693 | - |  | P14A C26 | 24 | 3475 | 3498 | 3497 | 2953 | - |
| M15A C45 | 25 | 183 | 748 | 963 | 689 | - |  | P14A C27 | 24 | 80 | 99 | 54 | 109 | - |
| M15A C48 | 25 | 3017 | 4197 | 4134 | 774 | - |  | P14B C48 | 24 | 66 | 63 | 616 | 404 | - |
| M15B C53 | 25 | 2917 | 479 | 2927 | 4058 | + |  | P14B C28 | 24 | 552 | 927 | 1065 | 225 | - |
| M15B C57 | 25 | 704 | 2581 | 409 | 818 | + |  | P14B C52 | 24 | 552 | 927 | 1065 | 225 | - |
| M15C C13 | 25 | 2230 | 5908 | 5512 | 4114 | + |  | P14C C1 | 24 | 159 | 259 | 407 | 887 | - |
| M15C C14 | 25 | 3453 | 471 | 782 | 430 | - |  | P14C C2 | 24 | 158 | 261 | 755 | 493 | - |
| M15C C15 | 25 | 582 | 2306 | 2437 | 1859 | - |  | P14C C5 | 24 | 415 | 5500 | 532 | 1114 | - |
| GL-8 |  | 1 | 1 | 1 | 1 | - |  | GL-8 |  | 3 | 3 | 2 | 1 | - |
| B2542 |  | 1 | 1 | 1 | 1 | + |  | B2542 |  | 1 | 1 | 1 | 1 | + |
|  | PNGS | A | B | C | D |  |  |  | PNGS | A | B | C | D |  |
| M46A C73 | 23 | 2 | 3 | 4 | 3 | - |  | P13A C71 | 25 | 3 | 10 | 19 | 28 | + |
| M46A C74 | 23 | 7 | 6 | 7 | 10 | - |  | P13B C82 | 25 | 4 | 202 | 182 | 82 | - |
| M46A C75 | 23 | 9 | 7 | 8 | 9 | - |  | P13C C25 | 25 | 4 | 202 | 182 | 82 | - |
| M46B C82 | 23 | 6 | 10 | 5 | 11 | - |  | P13C C26 | 25 | 4 | 202 | 182 | 82 | - |
| M46B C83 | 23 | 8 | 10 | 9 | 9 | + |  | P13A C72 | 25 | 4 | 202 | 182 | 82 | - |
| M46C C48 | 23 | 9 | 5 | 10 | 7 | - |  | P13A C74 | 25 | 6 | 15 | 22 | 30 | - |
| M46C C49 | 23 | 6 | 5 | 9 | 7 | - |  | P13B C81 | 25 | 1 | 8 | 11 | 15 | - |
| M46C C50 | 23 | 9 | 8 | 7 | 8 | - |  | P13B C84 | 25 | 1 | 7 | 12 | 14 | - |
| GL-8 |  | 1 | 1 | 1 | 1 | - |  | P13C C27 | 25 | 1 | 1 | 1 | 1 | - |
| B2542 |  | 1 | 1 | 1 | 1 | + |  | GL-8 |  | 1 | 3 | 4 | 4 | - |
|  | PNGS | A | B | C | D |  |  | B2542 |  | 1 | 1 | 2 | 2 | + |
| M25A C100 | 25 | 2 | 92 | 787 | 782 | +/- |  |  | PNGS | A | B | C | D |  |
| M25A C98 | 25 | 579 | 890 | 3892 | 3321 | + |  | M14A C254 | 25 | 1 | 1 | 15 | 1258 | - |
| M25A C99 | 25 | 682 | 2390 | 3205 | 1610 | + |  | M14A C262 | 25 | 1 | 0 | 12 | 1813 | - |
| M25C C37 | 25 | 127 | 164 | 674 | 934 | - |  | M14A C267 | 25 | 1 | 1 | 16 | 887 | + |
| M25C C38 | 25 | 6 | 4 | 4 | 5 | - |  | M14B C20 | 25 | 0 | 1 | 25 | 3405 | - |
| M25C C39 | 24 | 6971 | 5261 | 4074 | 4602 | - |  | M14C C181 | 25 | 1 | 1 | 12 | 2320 | - |
| GL-8 |  | 1 | 1 | 1 | 1 | - |  | M14C C183 | 25 | 1 | 1 | 14 | 891 | - |
| B2542 |  | 2 | 1 | 1 | 1 | + |  | M14C C184 | 25 | 1 | 1 | 35 | 592 | - |
|  | PNGS | A | B | C | D |  |  | M14C C64 | 25 | 1 | 1 | 16 | 1186 | - |
| M31A C253 | 22 | 64 | 72 | 332 | NA | - |  | M14C C65 | 25 | 1 | 1 | 10 | 1801 | - |
| M31C C1 | 22 | 1 | 1 | 1 | NA | - |  | M14C C7 | 25 | 1 | 1 | 19 | 990 | - |
| M31C C10 | 21 | 1 | 1 | 1 | NA | - |  | GL-8 |  | 1 | 1 | 1 | 1 |  |
| M31C C11 | 22 | 1 | 1 | 1 | NA | + |  | B2542 |  | 1 | 1 | 1 | 1 |  |
| M31C C12 | 22 | 43 | 50 | 137 | NA | - |  |  | PNGS | A | B | C | D |  |
| M31C C13 | 22 | 1 | 1 | 1 | NA | + |  | M30A C39 | 25 | 1 | 1 | 2002 | 118 | - |
| M31C C14 | 22 | 60 | 48 | 213 | NA | + |  | M30B C29 | 25 | 1 | 1 | 622 | 109 | - |
| M31C C15 | 22 | 51 | 47 | 176 | NA | - |  | M30B C13 | 25 | 1 | 1 | 622 | 109 | - |
| M31C C16 | 22 | 12 | 14 | 37 | NA | - |  | M30B C8 | 25 | 1 | 1 | 622 | 109 | - |
| M31C C167 | 22 | 1 | 1 | 1 | NA | + |  | M30B C1 | 25 | 1 | 1 | 622 | 109 | - |
| M31C C17 | 22 | 1 | 1 | 1 | NA | + |  | M30A C40 | 25 | 1 | 1 | 622 | 109 | - |
| M31C C2 | 22 | 1 | 1 | 1 | NA | - |  | M30A C41 | 25 | 1 | 1 | 502 | 183 | - |
| M31C C4 | 23 | 17 | 17 | 60 | NA | + |  | M30A C42 | 25 | 1 | 1 | 1893 | 75 | - |
| M31C C5 | 22 | 1 | 1 | 1 | NA | - |  | M30A C45 | 25 | 1 | 1 | 3921 | 148 | - |
| M31C C7 | 22 | 1 | 1 | 2 | NA | + |  | M30B C12 | 25 | 1 | 1 | 2168 | 85 | - |
| M31C C8 | 22 | 18 | 18 | 28 | NA | - |  | M30B C27 | 25 | 1 | 2 | 174 | 146 | - |
| M31C C9 | 22 | 1 | 1 | 1 | NA | - |  | M30B C30 | 25 | 1 | 1 | 927 | 260 | - |
| GL-8 |  | 1 | 1 | 2 | NA | - |  | M30B C31 | 25 | 1 | 1 | 2513 | 85 | - |
| B2542 |  | 1 | 1 | 1 | NA | + |  | M30B C4 | 25 | 1 | 1 | 2223 | 63 | - |
|  | PNGS | A | B | C | D |  |  | M30B C7 | 25 | 1 | 2 | 759 | 147 | - |
| P2B C17 | 24 | 1 | 2 | NA | NA | - |  | M30C C15 | 25 | 1 | 1 | 374 | 88 | - |
| P2B C18 | 24 | 32756 | 23812 | NA | NA | - |  | M30C C16 | 25 | 1 | 1 | 1911 | 95 | - |
| P2B C21 | 24 | 2 | 3 | NA | NA | - |  | M30C C88 | 25 | 1 | 1 | 1348 | 112 | - |
| P2B C24 | 24 | 530 | 3192 | NA | NA | - |  | M30C C91 | 25 | 1 | 1 | 3769 | 103 | - |
| P2B C4 | 24 | 1 | 3 | NA | NA | - |  | M30C C92 | 25 | 1 | 1 | 2555 | 75 | + |
| P2B C43 | 24 | 1 | 4 | NA | NA | - |  | GL-8 |  | 1 | 1 | 1 | 1 | - |
| P2B C44 | 24 | 2 | 3 | NA | NA | - |  | B2542 |  | 1 | 2 | 2 | 3 | + |
| P2B C52 | 24 | 2432 | 985 | NA | NA | - |  |  | PNGS | A | B | C | D |  |
| P2B C55 | 24 | 4 | 2 | NA | NA | - |  | M26A C109 | 23 | 12 | 17 | 6 | NA | + |
| P2B C59 | 24 | 6752 | 6031 | NA | NA | - |  | M26A C110 | 23 | 21 | 65 | 12 | NA | + |
| P2B C6 | 24 | 1573 | 4508 | NA | NA | - |  | M26A C112 | 23 | 46 | 101 | 30 | NA | - |
| P2B C64 | 24 | 2 | 3 | NA | NA | - |  | M26C C49 | 24 | 1 | 1 | 1 | NA | - |
| P2B C65 | 24 | 1015 | 4877 | NA | NA | - |  | M26C C50 | 23 | 24 | 17 | 12 | NA | - |
| P2B C3 | 24 | 2 | 3 | NA | NA | - |  | M26C C58 | 24 | 1 | 2 | 1 | NA | - |
| P2B C10 | 24 | 2 | 3 | NA | NA | - |  | GL-8 |  | 1 | 1 | 1 | NA | - |
| P2B C72 | 24 | 2 | 3 | NA | NA | - |  | B2542 |  | 1 | 1 | 1 | NA | + |
| GL-8 |  | 1 | 1 | NA | NA | - |  |  | PNGS | A | B | C | D |  |
| B2542 |  | 1 | 2 | NA | NA | + |  | P9A C19 | 24 | 4 | 7 | 8 | 5 | - |
|  | PNGS | A | B | C | D |  |  | P9A C20 | 23 | 6 | 9 | 11 | 5 | - |
| P4A C31 | 25 | 102 | 16 | 24 | 17 | + |  | P9A C22 | 24 | 8 | 12 | 12 | 7 | - |
| P4A C32 | 25 | 111 | 19 | 31 | 22 | + |  | P9C C49 | 24 | 8 | 11 | 12 | 6 | - |
| P4B C109 | 23 | 1 | 1 | 3 | 7 | - |  | P9C C52 | 24 | 5 | 7 | 8 | 6 | - |
| P4B C2 | 25 | 1 | 1 | 2 | 3 | - |  | GL-8 |  | 1 | 1 | 2 | 1 | - |
| P4B C3 | 24 | 1 | 1 | 3 | 6 | - |  | B2542 |  | 1 | 1 | 2 | 2 | + |
| P4C C20 | 24 | 2 | 1 | 1 | 1 | - |  |  | PNGS | A | B | C | D |  |
| GL-8 |  | 1 | 1 | 2 | 2 | - |  | M47A C135 | 19 | 1 | 2 | 1 | 11 | - |
| B2542 |  | 1 | 1 | 1 | 1 | + |  | M47A C139 | 22 | 1 | 1 | 5 | 987 | - |
|  | PNGS | A | B | C | D |  |  | M47A C144 | 22 | 1 | 1 | 6 | 926 | - |
| P5A C2 | 25 | 1 | 4 | 5 | 6 | + |  | M47B C90 | 22 | 1 | 1 | 10 | 1153 | - |
| P5B C118 | 26 | 0 | 2 | 4 | 3 | - |  | M47B C91 | 22 | 1 | 2 | 163 | 813 | - |
| P5B C3 | 25 | 1 | 4 | 5 | 5 | - |  | M47B C94 | 21 | 1 | 1 | 27 | 1634 | - |
| P5C C25 | 25 | 1 | 4 | 7 | 6 | - |  | M47C C65 | 22 | 1 | 1 | 8 | 994 | - |
| P5C C26 | 25 | 1 | 3 | 4 | 4 | - |  | M47C C68 | 22 | 1 | 1 | 7 | 957 | - |
| P5B C4 | 25 | 1 | 3 | 4 | 4 | - |  | GL-8 |  | 1 | 1 | 1 | 1 | - |
| P5A C5 | 25 | 1 | 3 | 4 | 4 | - |  | B2542 |  | 1 | 1 | 1 | 1 | + |
| P5C C27 | 25 | 1 | 3 | 4 | 4 | - |  |  | PNGS | A | B | C | D |  |
| GL-8 |  | 1 | 1 | 1 | 1 | - |  | M49A C85 | 25 | 1 | 1 | 0 | 0 | - |
| B2542 |  | 1 | 1 | 1 | 1 | + |  | M49A C86 | 25 | 1 | 1 | 1 | 1 | - |
|  | PNGS | A | B | C | D |  |  | M49A C87 | 23 | 88 | 167 | 152 | 70 | - |
| P7B C50 | 23 | 2 | 3 | 3 | 5 | - |  | M49C C74 | 25 | 1 | 1 | 1 | 1 | - |
| P7C C61 | 22 | 2 | 2 | 2 | 8 | +/- |  | M49C C76 | 24 | 1 | 1 | 1 | 1 | - |
| GL-8 |  | 2 | 2 | 2 | 2 | - |  | M49C C77 | 25 | 1 | 1 | 1 | 1 | - |
| B2542 |  | 2 | 2 | 2 | 2 | + |  | GL-8 |  | 0.7 | 0.9 | 0.8 | 0.7 | - |
|  | PNGS | A | B | C | D |  |  | B2542 |  | 0.8 | 1.1 | 1 | 1.1 | + |
| P8A C10 | 24 | 3 | 4 | 3 | NA | - |  |  | PNGS | A | B | C | D |  |
| GL-8 |  | 3 | 5 | 4 | NA | - |  | P11B C35 | 25 | 2 | 3 | 3 | 2 | - |
| B2542 |  | 8 | 15 | 14 | NA | + |  | P11B C40 | 25 | 2 | 3 | 3 | 2 | - |
|  | PNGS | A | B | C | D |  |  | P11B C46 | 25 | 2 | 3 | 3 | 2 | - |
|  |  |  |  |  |  |  |  | P11C C87 | 25 | 2 | 3 | 3 | 2 | - |
| P22C C73 | 23 | 6217 | 14833 | 5678 | NA | - |  | P11C C89 | 25 | 2 | 3 | 3 | 2 | - |
| P22C C80 | 24 | 54080 | 31011 | 9650 | NA | - |  | P11C C90 | 25 | 2 | 3 | 3 | 2 | - |
| GL-8 |  | 5 | 7 | 5 | NA | - |  | P11C C88 | 25 | 5 | 6 | 7 | 6 | - |
| B2542 |  | 3 | 4 | 4 | NA | + |  | GL-8 |  | 5 | 9 | 9 | 5 | - |
|  | PNGS | A | B | C | D |  |  | B2542 |  | 4 | 7 | 8 | 5 | + |
| M2C C1 | 20 | 4 | 4 | 277 | 69 | +/- |  |  | PNGS | A | B | C | D |  |
| M2C C5 | 20 | 2 | 1 | 6 | 4 | - |  | P18B C13 | 25 | 3028 | 3084 | 2562 | NA | - |
| GL-8 |  | 1 | 1 | 1 | 1 | - |  | GL-8 |  | 7 | 10 | 6 | NA | - |
| B2542 |  | 1 | 1 | 1 | 1 | + |  | B2542 |  | 2 | 2 | 2 | NA | + |
|  | PNGS | A | B | C | D |  |  |  | PNGS | A | B | C | D |  |
| M44A C91 | 24 | 13 | NA | NA | NA | - |  | M50A C296 | 23 | 1 | NA | NA | NA | - |
| M44A C92 | 25 | 18 | NA | NA | NA | - |  | M50A C294 | 23 | 1 | NA | NA | NA | - |
| GL-8 |  | 1 | NA | NA | NA | - |  | GL-8 |  | 1 | NA | NA | NA | - |
| B2542 |  | 1 | NA | NA | NA | + |  | B2542 |  | 1 | NA | NA | NA | + |
|  | PNGS | A | B | C | D |  |  |  | PNGS | A | B | C | D |  |
| M3A C168 | 24 | 3 | NA | NA | NA | - |  | P21B C75 | 24 | 1 | 2 | 2 | NA | - |
| GL-8 |  | 1.6 | NA | NA | NA | - |  | P21B C76 | 24 | 1 | 2 | 2 | NA | - |
| B2542 |  | 4 | NA | NA | NA | + |  | P21B C77 | 24 | 1 | 2 | 2 | NA | - |
|  | PNGS | A | B | C | D |  |  | P21B C79 | 24 | 1 | 2 | 2 | NA | - |
| M33A C146 | 23 | 1 | 1 | NA | NA | + |  | P21C C51 | 22 | 1 | 2 | 2 | NA | - |
| M33A C147 | 24 | 2 | 1 | NA | NA | + |  | P21C C52 | 22 | 2 | 2 | 3 | NA | - |
| M33A C150 | 23 | 2 | 1 | NA | NA | + |  | GL-8 |  | 2 | 3 | 2 | NA | - |
| M33A C152 | 23 | 2 | 1 | NA | NA | + |  | B2542 |  | 2 | 2 | 2 | NA | + |
| M33A C153 | 23 | 2 | 1 | NA | NA | - |  |  | PNGS | A | B | C | D |  |
| M33A C155 | 23 | 2 | 1 | NA | NA | + |  | M28A C70 | 25 | 1 | 2 | 1 | 2 | - |
| M33B C36 | 23 | 2 | 2 | NA | NA | + |  | M28A C74 | 24 | 1 | 1 | 1 | 1 | - |
| M33B C37 | 23 | 2 | 2 | NA | NA | + |  | M28A C79 | 25 | 1 | 2 | 1 | 2 | - |
| M33B C39 | 23 | 1 | 2 | NA | NA | + |  | M28B C62 | 25 | 1 | 1 | 1 | 1 | - |
| M33B C41 | 23 | 2 | 2 | NA | NA | - |  | M28B C63 | 25 | 1 | 1 | 1 | 2 | - |
| M33B C42 | 23 | 1 | 2 | NA | NA | + |  | M28B C65 | 25 | 1 | 1 | 1 | 1 | - |
| M33B C43 | 23 | 2 | 1 | NA | NA | + |  | M28B C66 | 25 | 1 | 2 | 1 | 2 | - |
| M33B C44 | 23 | 2 | 1 | NA | NA | + |  | M28B C67 | 25 | 1 | 1 | 1 | 1 | - |
| M33B C45 | 23 | 1 | 1 | NA | NA | + |  | M28C C75 | 25 | 1 | 1 | 1 | 1 | - |
| M33B C46 | 23 | 2 | 1 | NA | NA | + |  | M28C C76 | 25 | 1 | 1 | 1 | 1 | - |
| M33B C52 | 24 | 1 | 2 | NA | NA | + |  | M28C C77 | 25 | 1 | 1 | 1 | 1 | - |
| M33B C54 | 23 | 2 | 1 | NA | NA | + |  | GL-8 |  | 1 | 1 | 1 | 1 | - |
| M33B C62 | 23 | 2 | 1 | NA | NA | + |  | B2542 |  | 1 | 1 | 1 | 1 | + |
| M33B C64 | 23 | 1 | 1 | NA | NA | + |  |  | PNGS | A | B | C | D |  |
| GL-8 |  | 1 | 1 | NA | NA | - |  | M8C C144 | 21 | 1 | 1 | 1 | 1 | - |
| B2542 |  | 1 | 1 | NA | NA | + |  | GL-8 |  | 1 | 2 | 2 | 2 |  |
|  | PNGS | A | B | C | D |  |  | B2542 |  | 1 | 1 | 1 | 1 |  |
| M11A C250 | 23 | 1 | 1 | 1 | NA | - |  |  | PNGS | A | B | C | D | - |
| M11A C242 | 22 | 1 | 1 | 1 | NA | + |  | M29C C1 | 25 | 1 | 1 | 1 | 1 | - |
| M11A C238 | 23 | 0 | 1 | 1 | NA | - |  | M29C C2 | 25 | 1 | 1 | 1 | 1 | - |
| M11B C53 | 23 | 1 | 1 | 1 | NA | - |  | M29C C52 | 25 | 1 | 1 | 1 | 1 | - |
| M11B C94 | 23 | 1 | 1 | 1 | NA | - |  | GL-8 |  | 1 | 1 | 1 | 1 | - |
| M11B C96 | 23 | 0 | 0 | 0 | NA | - |  | B2542 |  | 1 | 1 | 1 | 1 | + |
| M11C C150 | 23 | 1 | 1 | 1 | NA | - |  |  | PNGS | A | B | C | D |  |
| M11C C82 | 23 | 1 | 1 | 1 | NA | - |  | M32A C159 | 25 | 1 | 1 | 1 | 1 | - |
| M11C C7 | 23 | 1 | 1 | 1 | NA | - |  | M32A C160 | 25 | 1 | 1 | 1 | 1 | - |
| M11C C168 | 23 | 1 | 1 | 1 | NA | - |  | M32A C161 | 25 | 1 | 1 | 1 | 1 | - |
| M11C C151 | 23 | 1 | 1 | 1 | NA | - |  | GL-8 |  | 1 | 1 | 1 | 1 | - |
| M11C C152 | 23 | 1 | 1 | 1 | NA | - |  | B2542 |  | 1 | 1 | 1 | 1 | + |
| M11C C78 | 23 | 1 | 1 | 1 | NA | - |  |  | PNGS | A | B | C | D |  |
| M11C C164 | 23 | 1 | 1 | 1 | NA | - |  | P17B C94 | 24 | 1 | 1 | 1 | NA | - |
| M11C C1 | 23 | 1 | 1 | 1 | NA | + |  | P17A C100 | 24 | 1 | 1 | 1 | NA | - |
| GL-8 |  | 1 | 1 | 1 | NA | - |  | P17A C99 | 23 | 1 | 1 | 1 | NA | - |
| B2542 |  | 1 | 1 | 1 | NA | + |  | P17B C1 | 24 | 1 | 1 | 1 | NA | - |
|  | PNGS | A | B | C | D |  |  | P17B C3 | 24 | 1 | 1 | 1 | NA | - |
| M16AC130 | 24 | 1 | 1 | 1 | NA | +/- |  | P17B C93 | 24 | 1 | 1 | 1 | NA | - |
| M16AC220 | 24 | 1 | 1 | 1 | NA | +/- |  | P17B C95 | 24 | 1 | 1 | 1 | NA | - |
| M16AC215 | 24 | 1 | 1 | 1 | NA | +/- |  | P17C C43 | 24 | 1 | 1 | 1 | NA | - |
| M16AC209 | 24 | 1 | 1 | 1 | NA | +/- |  | P17C C48 | 24 | 1 | 1 | 1 | NA | - |
| M16C C30 | 24 | 1 | 1 | 1 | NA | + |  | GL-8 |  | 1 | 1 | 1 | NA | - |
| M16C C32 | 24 | 1 | 1 | 1 | NA | +/- |  | B2542 |  | 1 | 1 | 1 | NA | + |
| M16C C80 | 24 | 1 | 1 | 1 | NA | +/- |  |  | PNGS | A | B | C | D |  |
| GL-8 |  | 1 | 1 | 1 | NA | - |  | M5A C14 | 22 | 1 | 1 | 1 | NA | + |
| B2542 |  | 1 | 1 | 1 | NA | + |  | M5A C15 | 22 | 1 | 1 | 1 | NA | - |
|  | PNGS | A | B | C | D |  |  | M5A C17 | 22 | 1 | 1 | 1 | NA | + |
| M20A C284 | 23 | 1 | 1 | NA | 1 | - |  | M5B C62 | 23 | 1 | 1 | 1 | NA | - |
| GL-8 |  | 1 | 1 | NA | 1 | - |  | M5B C68 | 22 | 1 | 1 | 1 | NA | - |
| B2542 |  | 1 | 1 | NA | 1 | + |  | M5C C21 | 23 | 1 | 1 | 1 | NA | - |
|  | PNGS | A | B | C | D |  |  | M5C C22 | 23 | 1 | 1 | 1 | NA | - |
| P6B C3 | 24 | 1 | 1 | 1 | 1 | - |  | M5C C28 | 23 | 1 | 1 | 1 | NA | - |
| P6A C68 | 24 | 1 | 1 | 1 | 1 | **-** |  |  | PNGS | A | B | C | D |  |
| PA C69 | 24 | 1 | 1 | 1 | 1 | **-** |  | M41A C120 | 22 | 1 | 1 | 1 | NA | + |
| P6A C70 | 24 | 1 | 1 | 1 | 1 | **-** |  | M41A C122 | 22 | 1 | 1 | 1 | NA | +/- |
| P6B C1 | 24 | 1 | 1 | 1 | 1 | **-** |  | M41C C32 | 22 | 1 | 1 | 1 | NA | - |
| P6B C2 | 24 | 1 | 1 | 1 | 1 | **-** |  | M41C C34 | 22 | 1 | 1 | 1 | NA | - |
| P6C C22 | 24 | 1 | 1 | 1 | 1 | **-** |  | M41C C39 | 22 | 1 | 1 | 1 | NA | +/- |
| P6C C63 | 24 | 1 | 1 | 1 | 1 | **-** |  | M41C C66 | 22 | 1 | 1 | 1 | NA | - |
| GL-8 |  | 1 | 1 | 1 | 1 | **-** |  | M41C C67 | 22 | 1 | 1 | 1 | NA | - |
| B2542 |  | 1 | 1 | 1 | 1 | + |  | M41C C72 | 22 | 1 | 1 | 1 | NA | - |
|  | PNGS | A | B | C | D |  |  | M41C C73 | 22 | 1 | 1 | 1 | NA | - |
| M1A C3 | 23 | 1 | 1 | 1 | 1 | - |  | M41C C82 | 22 | 1 | 1 | 1 | NA | - |
| M1C C100 | 23 | 1 | 1 | 1 | 1 | - |  | M41C C87 | 22 | 1 | 1 | 1 | NA | - |
| M1C C97 | 23 | 1 | 1 | 1 | 1 | - |  | GL-8 |  | 1 | 1 | 2 | NA | - |
| GL-8 |  | 1 | 1 | 1 | 1 | - |  | B2542 |  | 1 | 1 | 1 | NA | + |
| B2542 |  | 1 | 1 | 1 | 1 | + |  |  |  |  |  |  |  |  |
