# Supplementary material for: Emergence of CD134 cysteine-rich domain 2 (CRD2)-independent strains of feline immunodeficiency virus (FIV) is associated with disease progression in naturally infected cats
Source: Retrovirology. 2014 Nov 28;11:95. doi: 10.1186/s12977-014-0095-7 (PMC4275942; doi:10.1186/s12977-014-0095-7)
Supplement: Additional file 4: Figure S1. — PNGS at the stem of V5 region of Env is associated with distinct mode of receptor utilisation. [file 12977_2014_95_MOESM4_ESM.docx]

**Additional file 4: Figure S1** PNGS at the stem of V5 region of Env is associated with distinct receptor utilisation. Receptor utilisation by M11A C242 WT was compared with mutants E838 and T520. To confirm the role of the PNGS at position 520, receptor usage of M11A C164 was compared with that of mutant A520. GL8 and B2542 were tested in parallel as representative CRD2-dependent and -independent pseudotypes respectively. Each bar represents mean luciferase activity (cpm) ± standard error (n=3).
